# Image-Based Dietary Assessment Ability of Dietetics Students and Interns

**DOI:** 10.3390/nu9020114

**Published:** 2017-02-07

**Authors:** Erica Howes, Carol J. Boushey, Deborah A. Kerr, Emily J. Tomayko, Mary Cluskey

**Affiliations:** 1 College of Public Health and Human Sciences, Nutrition, Oregon State University, 101 Milam Hall, Corvallis, OR 97331-3303, USA; erica.howes@oregonstate.edu (E.H.); emily.tomayko@oregonstate.edu (E.J.T.); 2 Epidemiology Program, University of Hawaii Cancer Center, Honolulu, HI 96813, USA; cjboushey@cc.hawaii.edu; 3 School of Public Health, Curtin University, Perth, WA 6102, Australia; d.kerr@curtin.edu.au

**Keywords:** dietary assessment, image-based dietary records, dietetics education, serving-size estimation

## Abstract

Image-based dietary assessment (IBDA) may improve the accuracy of dietary assessments, but no formalized training currently exists for skills relating to IBDA. This study investigated nutrition and dietetics students’ and interns’ IBDA abilities, the training and experience factors that may contribute to food identification and quantification accuracy, and the perceived challenges to performing IBDA. An online survey containing images of known foods and serving sizes representing common American foods was used to assess the ability to identify foods and serving sizes. Nutrition and dietetics students and interns from the United States and Australia (*n* = 114) accurately identified foods 79.5% of the time. Quantification accuracy was lower, with only 38% of estimates within ±10% of the actual weight. Foods of amorphous shape or higher energy density had the highest percent error. Students expressed general difficulty with perceiving serving sizes, making IBDA food quantification more difficult. Experience cooking at home from a recipe, frequent measuring of portions, and having a food preparation or cooking laboratory class were associated with enhanced accuracy in IBDA. Future training of dietetics students should incorporate more food-based serving size training to improve quantification accuracy while performing IBDA, while advances in IBDA technology are also needed.

## 1. Introduction

Current dietary assessment methods, including dietary records, 24-h dietary recalls, and food frequency questionnaires, are subject to errors that can influence overall accuracy of the assessment. Validation studies using doubly-labeled water have revealed high rates of underreporting using traditional diet assessment methods, especially in certain subpopulations [1,2]. In other cases, overreporting has been observed in cultures in which abundance of food is considered an indicator of status or wealth [3]. One method of preventing errors in reporting is training participants on serving size estimation skills. Several studies have reported improved accuracy after training individuals; however, in comparison to doubly-labeled water, accuracy remains low [4,5,6,7,8].

In recent years, image-based dietary assessment methods have emerged as potential tools for increasing the accuracy of reported diet intake [9,10]. Images may be captured actively by the participant, using a camera or smartphone, or taken passively by a wearable device. Images may be used to complement retrospective methods of diet assessment (image-assisted dietary assessment, IADA) [11]. The use of images allows for visual representations of the client’s diet to be captured, removing some of the reliance on memory and providing a more objective measure of a client’s diet [12]. In certain settings, such as patients seen exclusively via telehealth (provision of healthcare services remotely using technology), images may replace other recording methods, relying entirely on the images [13,14]. Limited data indicate that dietitians with five or more years of practice experience can effectively use images to measure dietary intake; however, their use is relatively novel in dietetics practice [15].

In response to the increased use of IBDA, dietetics training will need to incorporate specific learning experiences or teaching strategies that promote enhanced accuracy in interpretation of images for assessing the diets of clients. However, the skill level and accuracy of current dietetics students and interns at using digital images of food is unknown. The objectives of this study were (1) to assess the ability of nutrition and dietetics students and interns to perform IBDA; (2) to explore factors relating to dietetics training and experience that may contribute to enhanced accuracy in IBDA; and (3) to identify challenges to performing IBDA. We hypothesized that years of formal dietetics training and experience performing traditional methods of dietary intake assessment would be associated with a higher degree of accuracy in performing IBDA.

## 2. Materials and Methods

### 2.1. Needs Assessment

A sample of dietitians and other nutrition professionals with formal training in the field of dietetics and currently using food images as part of their practice were identified and surveyed using a brief qualitative survey about factors that may affect the accuracy of food identification and quantification through images. Because little research has explored these factors, qualitative research was used as a starting point for identifying potential explanatory variables relating to identification and quantification ability. Their responses to open-ended questions about their knowledge, skills, and experiences were recorded and downloaded from an online survey platform. Two researchers independently read the survey responses, seeking areas of agreement and commonality expressed by the respondents. Researchers then met to compare their interpretation of the responses and developed major themes from each area of interest. Themes were then used to develop response choices for the questions in the Diet Assessment Survey.

### 2.2. Diet Assessment Survey

#### 2.2.1. Participants

A cross-sectional survey was used to assess dietetics students and interns’ abilities to interpret images of food and factors that influenced this ability. A convenience sample of third- and fourth-year undergraduate students or graduate students in nutrition or dietetics and dietetic interns from three land grant institutions in the United States and one large institution in Australia were recruited via email during the summer and fall of 2016. Recruitment sites were chosen based on the availability of participants and access to fiducial markers (FMs) a small reference object used for interpretation of the color and size of the foods in the images [16,17,18]. Individuals were required to be 18 years or older and students in a nutrition or related program. A minimum sample size of approximately 30 students was estimated for sufficient power based on previous similar studies [19,20], with a desired distribution of students from each institution to minimize bias based on similar background and training. The study was approved by the Oregon State University Institutional Review Board (Approval #7332). Written informed consent was obtained from all participants prior to starting the survey, and all consented participants received a fiducial marker.

#### 2.2.2. Diet Assessment Survey

**Figure 1:**
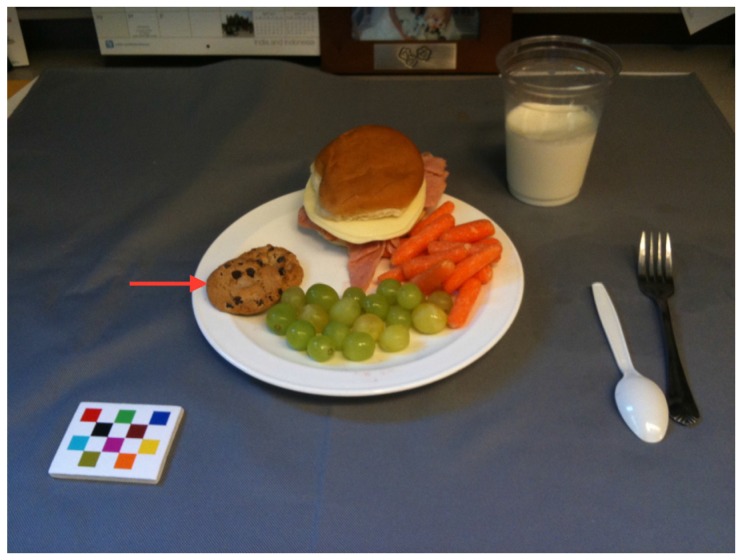
Example of an image shown to participants for the identification serving of the Diet Assessment Survey.

Participants completed an online survey prepared with Qualtrics (Provo, UT, USA) in which they were shown images of plated food (example, Figure 1). Food items were prepared in a kitchen using standardized recipes, thus the quantities and identities of the foods in the images were known (ground truth). Foods in the images corresponded to existing entries in the United States Department of Agriculture (USDA) Food and Nutrient Database for Dietary Studies (FNDDS). All of the images contained a FM as used with the mobile food record as a reference object for size and color, aimed to reduce bias introduced by the use of different screens in using the images [21]. Specific foods were chosen based on food consumption data from the National Health and Nutrition Examination Survey (NHANES) to reflect commonly consumed foods in the United States and to reflect a variety of food groups and food forms.

First, the participants were tested on their ability to identify nine foods (provolone cheese, white roll, deli ham, chocolate chip cookie, 100% whole wheat bread, raw tomatoes, hard pretzels, apple juice, soda) in a randomly presented set of images using the *What’s in the Foods You Eat* search tool embedded within the online survey. The search tool is linked to the USDA Food and Nutrient Database for Dietary Studies (FNDDS) for nutrient information. Participants were given written and video instructions on how to navigate the search tool to minimize potential errors in identification due to low familiarity with the database. Participants recorded the name of the most appropriate food found in the database, along with its corresponding food code.

Next, participants estimated the quantity of foods in a second set of nine randomly presented images (100% whole wheat bread, banana, peanut butter, coffee, jelly, mixed salad greens, creamy dressing, 2% milk, potato chips). A variety of household measurement units and multiple measurement descriptors were available to choose from based on the food’s entry in the *What’s in the Foods You Eat Search Tool*. Participants were instructed to choose one unit to use based on personal preference, which then was converted into grams using the conversion factors found in FNDDS. At the conclusion of the survey, the participants received feedback on the accuracy of their answers. Participants were shown the images with the answer they provided, along with the ground truth answer for both identification and quantification. For quantification, the ground truth value was given in the same unit that the participant provided.

#### 2.2.3. Response to IBDA

Based on the information determined in the needs assessment, a survey was developed with a series of multiple-choice questions about challenges associated with identifying and quantifying foods from images. Respondents could select multiple answers, as applicable. The type of device that participants used to take the survey was also collected. Information was collected on age, gender, institutional affiliation, educational status classification, total years of higher education, assessment of familiarity with dietetics concepts, and dietary analysis software used. To assess formal dietetics training experiences, participants were asked the number of times they had performed specific nutrition and dietetics tasks or skills, such as standardizing recipes and using dietary analysis software. Answer choices ranged from “0–1 times” to “50+ times”, with an additional choice for “don’t know.” For some factors, a Likert-type scale was used to assess the participant’s self-efficacy at completing the task or skill. To assess other experiences outside of formal nutrition and dietetics training, participants were asked about the frequency with which they perform certain tasks such as grocery shopping, cooking with or without a recipe, or cooking in a foodservice operation and about their self-efficacy in identifying common foods associated with cultures other than their home country. Because of the novelty of the topic, validated questionnaires were not available for use.

#### 2.2.4. Data Analysis

Survey data were analyzed using RStudio (Version 0.99.842—© 2009–2016 RStudio, Inc., Boston, MA, USA). Data regarding demographics and experience information, benefits and challenges to performing IBDA, and experiences in and out of formal dietetics training were reported using frequencies and percentages.

*Identification of Foods from Images.* Answers provided by participants for identification of the foods in the images were coded as correctly identified if they met the guidelines as outlined in Schap et al. [19]. For participants who failed to enter the food code but entered a food description from FNDDS, the corresponding food code was used to code the response as correct or incorrect. The percent of foods correctly identified was computed for each participant, and the percent of times a food was correctly identified was computed for each food.

To account for differences in energy between similar foods such as varying fat contents of milk, ratio of energy (in kcals) in the identified food and energy present in the food in the image was computed using the known gram weight of the food in the image. For example, if 100 grams of skim milk (the ground truth value) was misidentified to be whole milk, the ratio of kcals present in 100 grams of whole milk would be divided by the kcals present in 100 grams of skim milk. A score <1 indicates underestimation of energy, and a score >1 indicates overestimation of energy. The average score was computed for each food product present in the survey to get a measure of the extent to which the misidentification of the food would influence energy estimates for a food record.

*Quantification of Foods from Images.* The conversion data in FNDDS was used to convert the estimates of serving size to a gram weight value. The gram weights of the foods were compared to the ground truth values for each food to obtain a value for estimation error. The number of participants who estimated the quantity within ±10% of the ground truth was calculated for each food. The estimated weights were divided by ground truth weights to obtain a ratio, with values <1 indicating underestimation and >1 indicating overestimation, similar to the process performed by Six et al. [22]. The average ratio was computed for each food item present in the survey.

To obtain the average accuracy in quantifying foods per person, the following formula was used to compute the percent error for the estimates for each food:

(1) $$\frac{\left|reported\;weight-ground\;truth\;weight\right|}{ground\;truth\;weight}\;\times \;100$$

The mean percent error was computed for each person across all foods estimated. Though this method did not account for under- or overestimations, it prevented underestimations and overestimations from canceling each other out and obscuring inaccuracies in estimation.

*Association of Education and Background Variables with Accuracy*. Multiple linear regression was used to determine variables predictive of accuracy in identification and quantification. Identification ability (percent of foods identified correctly) and quantification ability (average percent error) were used as the main outcome variables. Explanatory variables were selected for the regression based on the a priori hypotheses and the themes generated from the final open-ended question of the survey that asked about the perceived influence of experiences on image interpretation ability. The adjustment variables used in the model were university, age (continuous), years of education (as categories), and type of academic program. β values, 95% confidence intervals, and *p* values for the unadjusted and adjusted models were obtained using the lm command in R. Plots of residuals versus fitted values and normal quantile plots were used to check model assumptions. The significance level was set at α < 0.05. Likelihood ratio tests were used to test for interaction effects for age, university, and years of education.

## 3. Results

### 3.1. Needs Assessment

Four individuals from the United States and Australia provided survey responses. The panel identified specific knowledge and skills that aided in their ability to perform IBDA, as well as challenges with IBDA and benefits to using IBDA (Table 1).

### 3.2. Diet Assessment Survey

A total of 142 students consented to participate in the study; 114 students and interns completed the survey and were included in analysis. Demographic information for non-participants was not collected. Among participants included in analysis, 114 completed the food identification and quantification and 104 completed the post-survey. Participants were primarily identified as young adult (21–25 years, 65.8%) and female (88.60%). Most respondents were undergraduates in Nutrition and Health Science (40.35%), undergraduates in Dietetics (34.21%), and Dietetic Interns (9.65%). To complete the survey, 53% of participants used a desktop computer, 42% used a laptop computer, and 5% a tablet.

Of the foods tested for identification, participants were best able to identify the soft drink (97% identified correctly) and apple juice (92% identified correctly). The lowest number of correct identifications were the ham luncheon meat (48% identified correctly) and the whole wheat bread (65% identified correctly) (Table 2). The greatest difference in energy for the serving of food present between the foods identified by participants and the ground truth food was for the ham luncheon meat, with the misidentification leading to an average of 48 more kcals estimated than the actual kcal content. For all other foods in the identification portion of the survey, the mean kcals for the foods identified were similar to that of the ground truth kcals present in those foods (Table 2).

For the quantification portion of the survey, participants selected units of volume (cups, tablespoons, fluid ounces) more frequently than units of weight. Coffee and potato chips were the foods most commonly estimated using grams, with less than 14% of participants choosing grams to estimate the serving size. The salad, coffee, and ice cream had the fewest percentage of people quantifying the foods within ±10% of the ground truth weight at 0%, 9% and 18% of respondents, respectively. The foods quantified within ±10% of the ground truth weight by the most people were the banana, jelly, and creamy dressing, at 85%, 52% and 50%, respectively. The largest discrepancy in gram weight between the estimated serving and ground truth (1.89) and between the estimate and the energy (kcal) in the ground truth weight (93.7 kcals) was for the ice cream (Table 2).

Participants selected challenges with identifying and quantifying the foods based on images (Table 3). The most common challenge was poor image quality (reported by 51% of participants). Searching using the database was another common issue, with 51% of respondents reporting that “I couldn’t find the exact food I wanted” and 46% reporting that “it was difficult to know which foods to choose from the database”. In the “Other (please specify)” category, Australian students reported difficulty with knowing the differences between American and Australian words for foods quantification, 48% of participants reported general difficulty with serving size estimation, unrelated to the use of images. The angle at which the image was taken was also cited as a common problem in estimation (41%).

The results of the multiple linear regression are summarized in Table 4. Having food preparation and food lab experience significantly increased identification accuracy (*p* < 0.01). Experience interpreting food records did not lead to improvements in identification or quantification ability. Experience measuring volume related to improvements in identification ability, even with small increases in frequency from never to once per month or a few times per year (*p* < 0.01). In the adjusted model, only daily volume measurement led to a significant difference from never measuring volume (*p* = 0.05). There were no significant differences in identification or quantification ability based on the number of years of higher education. For identification ability, ANOVA analysis revealed interactions between the university attended and having had food lab experience, volume measurement experience, and foodservice cooking experience. For quantification ability, interaction effects were found between age and frequency of measuring by weight and cooking in a foodservice operation, and between university attended and the amount of experience interpreting food records.

## 4. Discussion

The current study demonstrates that nutrition and dietetics students and interns from the United States and Australia need more training to successfully identify foods from images and quantify them within a reasonable degree of accuracy and that experience in food preparation, measuring, and cooking from a recipe were associated with greater accuracy in identification and quantification. Moreover, we identified factors relating to their ability to successfully perform these assessments. Our findings correspond with studies involving adolescents and dietitians, who could successfully identify most foods from digital images; however, the current study also considered the additional step of entering the identifications into a nutrition database, which adds more potential for error in a full nutrient analysis [19]. Participants were challenged by subtle differences in the appearance of foods, thus commonly misidentifying the ham luncheon meat as fresh ham, and whole wheat bread as rye bread due to similarities in appearance of images of those foods.

Serving size estimations were generally less accurate than identification, with only three foods estimated within ±10% of the ground truth weight by a majority of the respondents. Overall, 38% of the estimates were within ±10% of the ground truth, which is higher than the results found by Japur and Diez-Garcia [20] using foods estimated in person, who found that only 18.5% of serving size estimates by nutrition students were within ±10% of the ground truth. Participants in the study of Japur and Diez-Garcia estimated serving sizes using grams or mL, thus the greater degree of options in household units in the present study may have aided in ease of estimating serving sizes [20]. However, in the case of the salad greens, the ground truth volume was 1.75 cups, and respondents tended to use increments of 0.5 cups to quantify foods, thus underestimating or overestimating the food slightly outside of the ±10% cutoff. Williamson et al. [23] found that three trained analysts were able to successfully estimate portion sizes of foods from digital images with a small degree of error, consistent with our findings using nutrition and dietetics students. However, the current study represents a greater range of individuals with diversity in training, providing more information about the type of training that leads to greater accuracy.

The impact of the serving size estimation errors on the kcal estimates was fairly low, with the kcal differences between the kcals in the estimated serving size and the kcals in the ground truth serving size ranging from −0.39 to 93.70 kcals. For example, though no students correctly quantified the salad within ±10% of the ground truth weight, the impact of the error on the kcal estimate was within 10 kcals due to the low energy density of the food. The foods chosen represented a variety of energy densities and serving sizes, two factors that could impact the magnitude of error in kcal estimations, as well as the likelihood of serving size estimation error [20]. Consistent with the findings of Japur and Diez-Garcia, the foods with the highest percent errors were foods with higher energy densities, specifically ice cream, potato chips, creamy dressing, and jelly. These foods are also foods to limit based on the Dietary Guidelines for Americans and for which there is no recommended intake level or serving size. Peanut butter, though it had a relatively high energy density, was underestimated by research participants, potentially reflective of its reputation as a health-promoting food [24]. Foods with the highest percent error also tended to be amorphous foods, which often take the shape of the container they are in and are known to produce less accurate potion size estimations [4,6].

One difficulty experienced by participants completing the survey was ambiguity in the size units (including package size terminology) provided by FNDDS to quantify foods. Though several units were provided, 36% of students reported that, “the units provided were not what I would use to quantify the food.” Addressing regional and cultural differences and manufacturers’ differences in portion unit terminology for quantifying foods or food packages also may play a role in the successful quantification of foods using a database such as FNDDS. The container in which the food was presented may have also impacted estimates, such as the coffee, which was only quantified correctly by 9% of respondents. Though coffee is commonly consumed worldwide, it was presented in a clear, unlabeled plastic cup, an atypical vessel for serving a hot beverage.

An issue that may not be addressed by training is the ability of trained analysts to accurately estimate serving sizes from images. With advances in technology, automated image analysis may better address this issue. Lee et al. used image analysis to estimate portion weights of eating occasions captured by adolescents over a 24-h period. [25]. The results showed the automated estimates were more accurate than the estimates by adolescents. The authors concluded the ability of humans to estimate portion sizes remains a problem.

The foods tested reflected commonly consumed American foods; therefore, the Australian students may have been less familiar with the foods in the survey. Twenty-nine percent of respondents reported that “the foods in the images did not look like foods I was familiar with”, which may have been due to cultural differences between American and Australian foods. While the mean identification score for the Australian students was lower than the American students (78% correct vs. 83% correct), the difference was not statistically significant (*p* = 0.11). Fewer than 10% of survey respondents stated that they felt “very confident” in their ability to identify common foods from cultures other than their own, with only 29% reporting that they felt “confident”. These results further support the idea that greater cultural competency training is needed among dietetics students, particularly as it relates to common foods and patterns relevant to dietary assessment [26].

This work further underscores the need for training of research participants when using traditional forms of dietary intake assessment such as dietary recalls or food records. Based on our findings, there are several areas where training may improve the accuracy of assessment using images. First, quantification is the area in which dietetics students could most improve to enhance the accuracy of dietary intake assessments performed using images, particularly among amorphous foods and foods with high energy densities. Second, integrating more training around food preparation, measuring, and cooking from a recipe may enhance the ability of dietetics students to identify and quantify foods from digital images. The influence of the food lab on quantification ability is consistent with the work of Weber et al. [8] and Slawson and Eck [6], which found that training using food or food models led to greater accuracy in portion size estimations. Culinary training or food labs are possibilities for educational interventions to enhance serving size estimation [27]. The interaction effects observed between the university attended and many of the experiential factors supports the idea that differences in the nutrition and dietetics curriculum can result in significant differences in the ability of students to perform IBDA accurately. Moreover, based on the degree of error observed with nutrition and dietetics students who have significant experience with food and dietary analysis, an untrained research participant or patient would be likely to exceed that level of error, compromising the accuracy of a dietary assessment. Future work may examine the accuracy of untrained participants as a control group to better quantify the impact of any nutrition training on accuracy of IBDA.

Many of the challenges participants reported with conducting IBDA in this study were technology related issues, including difficulty making choices and finding exact foods within the USDA Food and Nutrition Dietary Database System (FNDDS). Other technology-related problems, like poor image quality, will likely be addressed in the future as the camera technology on mobile telephones continues to progress. For those photographing their food intake, more instruction in choosing the angle and other aspects of taking images of the food in the mobile food record (mFR) would easily resolve some issues.

A strength of this work was the number of participants surveyed in a novel area of investigation. However, a limitation of the present study was the small number of universities used for recruitment of participants, which may limit generalizability of findings to all dietetics students. Future studies may use a cross-section of universities more representative of dietetics students as a population. Another limitation was the reliance on online surveys completed remotely for data collection, which produced a low survey completion rate, as well as some errors with survey responses. For example, some participants used multiple units of measurement to quantify foods, limiting the interpretation of results, as the different units led to multiple estimates of portion size for a single food.

## 5. Conclusions

Our study demonstrated that having nutrition and dietetic training may facilitate accurate identification of foods via images. Our findings also suggest improvements that would facilitate IBDA include developments in the image related technologies necessary for successful IBDA, including photo quality and angle, device functionality for viewing images, and instructional strategies for optimizing dietary intake images by those taking photos for IBDA. With regard to estimation of serving sizes, we identified components of training or experiences that were associated with greater accuracy, which included hands-on training and direct experience in food preparation, particularly measuring and utilizing recipes and tools that emphasize learning about food portions and metrics. It is interesting to note that FNDDS use and familiarity was also related to IBDA accuracy. Therefore, searching dietary database training also should be addressed in nutrition and dietetics curricula. Using FNDDS was a reported challenge in identifying the correct food as well as in interpreting unit terminology and choosing serving size units in the database. Additionally, identifying how search algorithms in FNDDS may be a factor in accuracy of dietary assessments overall. Moreover, future research should be performed to test the efficacy of specific educational interventions to improve accurate perceptions of serving size from images, with an emphasis on estimating energy-dense amorphous foods.

## Figures and Tables

**Table 1:** Themes and representative quotes about image-based dietary intake assessment (IBDA) ability and challenges from a panel of four individuals with experience in completing IBDA.

Formal Dietetics Training		
Concept	Themes	Quotes
Skills relating to identification	Use of diet analysis software	“Using FoodWorks [diet analysis software] has helped gain a better understanding of common foods as well as experiences such as learning about the different dietary recall methods and the different types of foods people share when doing food records”
	Traditional dietary assessment methods	
	Analyzing menus	
	Knowledge of food patterns and combinations	
	Knowledge of food groups	
Skills relating to estimation	Measuring foods	“…an understanding of common household measuring cups and utensils as well as resources including books on portion size”
	Performing food conversions	
	Using portion size resources such as life-size images of food	
Knowledge relating to identification	Food group knowledge	“Dietetics training helped me to understand that not all foods are the same, therefore when I assess and identify foods I can do this is [sic] more detail”
	Nutritional differences between foods	
	Supermarket tours	
	Food classes	
Knowledge relating to estimation	Converting volumes to weights	“Understanding of common portion sizes and the way in which people may serve themselves food”
	Dietary guideline recommendations	
	Standard serving sizes for prepared/packaged foods	
	Typical serving sizes of the general public	
Experiences Outside of Formal Training		
Knowledge, skills, or personal experiences relating to identification	Cultural food preferences	“Different foods that I eat at home for religious festivals and occasions” “If you are not from Hawaii then it is difficult to identify some Hawaiian foods”
	Foodservice experience	
	Observation of eating habits	
	Grocery shopping	
	Cooking experience	
	Cultural food knowledge and awareness	
Knowledge, skills, or personal experiences relating to estimation	Weighing and measuring foods	“Cooking with specific measurements and estimating with that knowledge” “I cook frequently, therefore I understand how much of each ingredient goes into certain meal types.”
	Seeing measured portions	
	Food preparation and cooking	
	Recipe knowledge	
Challenges Using Images		
Identification	Poor image quality	“Some of the images were blurry which made it difficult” “Some of the foods did not look like typical versions”
	Unfamiliarity with foods in images	
	Foods blocked from view by other foods	
	Size of the screen	
	Inability to view all ingredients in mixed dishes or combination foods	
	Difficult to determine unseen components such as fat content of dairy products	
Estimation	Angle of the foods	“Couldn’t always see all the ingredients or all of the portion of ingredients/food”
	Placement of the FM	
	Obstruction of foods with other foods	
	Coding of the foods in the software	

**Table 2:** Participants (*n* = 114) ability to accurately identify and quantify selected foods from images in the Diet Assessment Survey.

**Food Identification**	***n***	**Identified Correctly ^a^**	**Kcal Ratio ^b^ (Response/Ground Truth)**		**Kcal Difference ^c^ (Response/Ground Truth)**	
	*n*	%	Mean	*σ*	Mean	*σ*
Ham, sliced, extra-lean, prepackaged or deli, luncheon meat ^d^	109	48	1.61	0.72	47.83	56.54
Bread, whole wheat, 100%	110	65	1.04	0.13	2.22	*7.82*
Cheese, provolone ^d^	112	66	1.01	0.16	1.27	22.77
Roll, white, soft ^d^	109	77	0.98	0.15	−1.97	18.71
Pretzels, hard	111	88	1.03	0.08	3.09	8.08
Cookie, chocolate chip	111	91	1.02	0.04	2.43	4.55
Tomatoes, raw	109	91	1.16	1.08	4.23	28.24
Apple juice	111	92	0.97	0.16	−4.39	22.93
Soft drink, cola-type	109	97	0.99	0.09	−1.69	19.2
**Food Quantification**	***n***	**Quantified Correctly ^e^**	**Weight Ratio ^f^**		**Kcal Difference ^c^**	
	*n*	%	Mean	*σ*	Mean	*σ*
Mixed salad greens, raw	93	0	0.89	0.28	−1.83	4.64
Coffee, NS as to type	92	9	0.87	0.29	−0.39	0.87
Ice cream, regular, flavors other than chocolate	92	18	1.89	0.75	93.7	79.67
Potato chips, regular cut	101	31	1.32	0.86	49.51	133.76
Peanut butter	96	43	0.86	0.32	−24.14	51.89
Milk, cow’s, fluid, 2%	99	47	0.92	0.47	−10.26	57.71
Creamy dressing	94	50	1.17	0.52	21.79	65.74
Jelly, all flavors	97	52	1.23	0.5	8.7	18.62
Banana, raw	97	85	1.08	0.25	7.74	24.25

^a^ Foods coded as correctly identified based on criteria in Schap [19]; ^b^ Energy (kcal) in food identified/calories in ground truth food; ^c^ Energy (kcal) in food identified or quantified–Energy (kcal) in ground truth food; ^d^ Foods contained in the same image, distinguished with labeled arrows; ^e^ Quantified within ±10% of ground truth weight; ^f^ (Reported grams)/(ground truth grams); NS = not specified.

**Table 3:** Perceived challenges experienced by participants (*n* = 104) in identifying foods from images using a database and estimating the serving sizes of foods in images.

Participant Post-Diet Assessment Survey Questions and Answers	*n*	%
What challenges did you experience using the What’s in the Foods You Eat Search Tool to code the foods in the images?		
I didn’t know the difference between the foods in the database	6	5.8
I couldn’t navigate the website easily	7	6.7
Other (please specify)	7	6.7
There were too many foods to choose from in the database	21	20.2
It took too long to find the food I was looking for	22	21.2
I didn’t know what search terms to use to find the food I wanted	30	28.9
It was difficult to know which food to choose from the database	48	46.2
I couldn’t find the exact food I wanted	53	51.0
After completing the image-assisted dietary assessment exercise and considering your results, what challenges did you experience while trying to identify the foods in the images, not related to the database?		
The way the foods were arranged made it difficult to see the foods	24	27.9
Poor image quality made it difficult to identify the foods	44	51.2
The foods in the images did not look like foods I was familiar with	25	29.1
The screen I was using was too small to see the image clearly	2	2.3
It was hard to tell what was in a mixed dish	16	18.6
Other (please specify)	15	17.4
After completing the image-assisted dietary assessment exercise and considering your results, what was the most challenging aspect of estimating the quantity of the foods in the images?		
The angle that the image was taken from made it hard to judge size	41	41.0
The fiducial marker (reference object) was not in a good place to help judge size	29	29.0
Other foods were in the way, which made judging quantity difficult	5	5.0
The units provided were not what I would use to quantify the food	36	36.0
General difficulty with size perception, unrelated to the image itself	48	48.0
Other (please specify)	7	7.0

**Table 4:** Linear regression analysis for experiences of participants relating to the ability to identify foods from digital images (percent of foods identified correctly) and quantify foods from digital images (average percent error).

Experience	Identification								Quantification							
	Unadjusted				Adjusted ^†^				Unadjusted				Adjusted ^†^			
	β	*p*	95% CI		β	*p*	95% CI		β	*p*	95% CI		β	*p*	95% CI	
Food preparation/cooking laboratory experience																
yes vs. no	0.21	<0.001	0.12	0.29	0.17	<0.001	0.07	0.28	−0.16	0.03	−0.29	−0.02	−0.13	0.09	−0.31	0.04
Performing dietary recalls																
2–5 times vs. 0–1 time	0.06	0.21	−0.04	0.16	0.07	0.29	−0.06	0.20	−0.08	0.33	−0.26	0.09	0.08	0.49	−0.15	0.31
5–10 times vs. 0–1 time	0.03	0.55	−0.08	0.15	0.07	0.34	−0.07	0.21	−0.15	0.14	−0.35	0.05	0.01	0.94	−0.24	0.26
11–50 times vs. 0–1 time	−0.01	0.90	−0.14	0.12	−0.01	0.88	−0.21	0.18	−0.13	0.27	−0.36	0.10	−0.07	0.68	−0.43	0.28
50+ times vs. 0–1 time	0.12	0.09	−0.02	0.26	−0.01	0.95	−0.27	0.25	−0.24	0.05	−0.48	0.00	−0.14	0.53	−0.60	0.31
Interpreting food records																
2–5 times vs. 0–1 time	−0.04	0.55	−0.17	0.09	−0.09	0.39	−0.28	0.11	−0.04	0.74	−0.25	0.18	0.05	0.75	−0.28	0.39
5–10 times vs. 0–1 time	−0.03	0.66	−0.17	0.11	−0.06	0.54	−0.24	0.13	−0.04	0.70	−0.27	0.18	0.07	0.68	−0.25	0.39
11–50 times vs. 0–1 time	0.02	0.79	−0.13	0.17	0.01	0.95	−0.23	0.24	0.05	0.69	−0.20	0.30	0.22	0.28	−0.18	0.61
50+ times vs. 0–1 time	0.00	1.00	−0.19	0.19	−0.13	0.43	−0.44	0.19	−0.19	0.22	−0.50	0.11	0.08	0.78	−0.46	0.62
Measuring portions using volume measurements (cups, tablespoons, teaspoons)																
once a month or a few x/year vs. never	0.54	<0.01	0.19	0.90	0.34	0.13	−0.10	0.77	−0.10	0.70	−0.64	0.43	0.05	0.89	−0.69	0.80
once a week or a few x/month vs. never	0.57	<0.01	0.23	0.91	0.38	0.07	−0.04	0.79	−0.10	0.70	−0.62	0.42	0.07	0.84	−0.64	0.78
daily or a few x/week vs. never	0.60	<0.001	0.27	0.94	0.41	0.05	−0.01	0.82	−0.21	0.42	−0.72	0.30	−0.03	0.94	−0.73	0.68
Measuring portions using weight measurements (grams, ounces)																
once a month or a few x/year vs. never	0.01	0.88	−0.16	0.19	0.10	0.41	−0.14	0.35	0.14	0.31	−0.13	0.40	0.24	0.25	−0.18	0.66
once a week or a few x/month vs. never	−0.07	0.45	−0.23	0.10	0.04	0.71	−0.18	0.27	0.08	0.54	−0.18	0.33	0.14	0.47	−0.25	0.53
daily or a few x/week vs. never	0.01	0.93	−0.16	0.17	0.11	0.33	−0.12	0.35	0.06	0.65	−0.19	0.31	0.07	0.72	−0.32	0.47
Cooking in a foodservice operation																
once a month or a few x/year vs. never	0.09	0.12	−0.02	0.20	0.05	0.48	−0.08	0.17	−0.03	0.72	−0.21	0.15	0.05	0.62	−0.16	0.27
once a week or a few x/month vs. never	−0.03	0.67	−0.15	0.10	−0.13	0.11	−0.29	0.03	0.04	0.67	−0.16	0.24	0.11	0.42	−0.16	0.38
daily or a few x/week vs. never	0.12	0.04	0.01	0.23	0.06	0.38	−0.08	0.21	0.15	0.11	−0.03	0.33	0.25	0.05	0.00	0.49
Cooking at home from a recipe																
once a month or a few x/year vs. never	−0.17	0.34	−0.53	0.19					−1.28	<0.001	−1.81	−0.75				
once a week or a few x/month vs. never	−0.24	0.19	−0.59	0.12	−0.01	0.84	−0.13	0.10	−1.22	<0.001	−1.74	−0.70	0.04	0.70	−0.16	0.23
daily or a few x/week vs. never	−0.28	0.31	−0.54	0.17	0.02	0.79	−0.11	0.14	−1.30	<0.001	−1.83	−0.78	−0.09	0.37	−0.30	0.12
Cooking at home without a recipe																
once a month or a few x/year vs. never	−0.02	0.88	−0.31	0.27	−0.05	0.76	−0.36	0.26	−0.06	0.78	−0.51	0.39	−0.11	0.64	−0.60	0.37
once a week or a few x/month vs. never	−0.15	0.27	−0.41	0.11	−0.11	0.42	−0.38	0.16	0.27	0.18	−0.13	0.67	0.24	0.27	−0.19	0.66
daily or a few x/week vs. never	−0.08	0.52	−0.33	0.17	−0.07	0.59	−0.33	0.19	0.12	0.55	−0.27	0.50	0.06	0.79	−0.35	0.46

^†^ variables adjusted to control for subject university, age (continuous), years of education (as categories), and type of academic program.
